# Stratification in health and survival after age 100: evidence from Danish centenarians

**DOI:** 10.1186/s12877-021-02326-3

**Published:** 2021-07-01

**Authors:** Jesús-Adrián Alvarez, Anthony Medford, Cosmo Strozza, Mikael Thinggaard, Kaare Christensen

**Affiliations:** 1 Interdisciplinary Centre on Population Dynamics, University of Southern Denmark, 5000 Odense, Denmark; 2 Danish Aging Research Center, Epidemiology, Biostatistics and Biodemography, Department of Public Health, University of Southern Denmark, Odense C, Denmark; 3 Department of Clinical Genetics and Department of Clinical Biochemistry and Pharmacology, Odense University Hospital, Odense, Denmark

**Keywords:** Heterogeneity, Centenarian, Survival, Health, Latent class analysis

## Abstract

**Background:**

The existence of a super-select group of centenarians that demonstrates increased survivorship has been hypothesized. However, it is unknown if this super-select group possesses similar characteristics apart from extreme longevity.

**Methods:**

In this study, we analyse high-quality health and survival data of Danish centenarians born in 1895, 1905 and 1910. We use Latent Class Analysis to identify unobserved health classes and to test whether these super-select lives share similar health characteristics.

**Results:**

We find that, even after age 100, a clear and distinct gradient in health exists and that this gradient is remarkably similar across different birth cohorts of centenarians. Based on the level of health, we identify three clusters of centenarians - robust, frail and intermediate - and show that these groups have different survival prospects. The most distinctive characteristic of the robust centenarians is the outperformance in different health dimensions (physical, functional and cognitive). Finally, we show that our health class categorizations are good predictors of the survival prospects of centenarians.

**Conclusions:**

There is a clear stratification in health and functioning among those over 100 years of age and these differences are associated with survival beyond age 100.

**Supplementary Information:**

The online version contains supplementary material available at 10.1186/s12877-021-02326-3.

## Background

Those who live to the oldest ages, particularly centenarians, are a select group [1]. Medford et al. [2] discuss the possibility of an additional layer of selection among centenarians – a so called “super-select” group – that consistently survives the longest beyond the age of 100 years. These individuals are the frontrunners of longevity, surviving as far as the 95^th^ percentile of the distribution of lifespans above age 100 (i.e. beyond age 105) [2] and they exhibit greater improvements in their individual lifespan than other centenarians [3, 4]. Though some may be robust from birth, resilience at younger ages does not necessarily translate into resilience during old age because an individual is exposed to the risk of sickness over their entire life course and may become infirm before reaching old age. It was previously believed that at extreme ages, survival chances were largely random and more driven by stochastic determinants than anything else [5]. However, Medford et al. [2] postulate that the super-select group of lives benefits most from improvements in medical technology and healthcare advances and are best positioned to take advantage of further increases in human lifespan. This hypothesis implies that (i) the super-select might share similar traits, (ii) such traits might be common across different birth cohorts and (iii) survival to extremely old ages may not be as random as some suggest. Therefore, a better understanding of the characteristics of exceptionally long-lived individuals may help to shed light on what is required for healthy aging.

Apart from extreme longevity, what traits distinguish the super-select? Centenarians have defeated death for at least 100 years, yet, no centenarian is exactly the same as another [1]. This uniqueness is due to different lifestyles [6–8], behaviour [9, 10], genetics [11], physiological make up [12–14], environmental determinants [15], exposure to prior and ongoing medical treatment [16–18] and many unobserved or unobservable factors [19] that ultimately lead to disparate lifespans. Most centenarians die within the first 2 years after reaching age 100 with relatively few surviving much longer^1^ [20]. Heterogeneity in the context of individual lifespans and, in both observed and unobserved traits, is therefore natural and common among centenarians [21]. This inherent heterogeneity entails that some centenarians will make it to the frontier of survival [22] by chance and not necessarily because of any traits that they have in common with the super-select [5]. Similarly, some might be categorized as super-select but will die soon after their 100^th^ birthday. Therefore, in order to correctly determine the traits of the super-select, it is paramount that the issue of heterogeneity is carefully addressed.

Previous studies on the health of nonagenarians (i.e. 93–95 years old) [23] provide valuable hints on the expected traits to be found in the super-select centenarians (i.e. 95^th^ percentile of the distribution of lifespans above age 100, beyond age 105 [2]). By using cluster analysis to control for heterogeneity in health, some researchers [24, 25] have shown that nonagenarians can be categorized according to specific health classes, where one class has a consistent advantage in relation to the others. It has also been shown that factors which are usually good at differentiating and predicting survival at younger ages (e.g. smoking, obesity level, education, number of chronic diseases) do not explain survival differences among nonagenarians [26]. Instead, cognitive and physical abilities and to some extent, an optimistic personality, are regarded as strong predictors [26–28]. Further, survival among nonagenarians is improving across cohorts [29]. These improvements are accompanied by better health and functioning across the health spectrum [30–33].

It cannot be taken for granted that the associations between health and survival previously shown for nonagenarians will automatically apply for those aged 100 or more. These associations [26, 30] cannot be blindly extrapolated to centenarians (or individuals surviving beyond age 100), because only 10–15% of nonagenarians make it to age 100 [20]. Furthermore, studies in Denmark and Sweden have shown that improvements in survival for centenarians are negligible when looking at the median and mean lifespan above age 100 [20]. Survival improvements for Denmark are observed for only a relatively small proportion, the super-select (i.e. the 95^th^ percentile of the distribution of lifespans above age 100, above age 105) [2] and are not present for Sweden. Therefore, the assessment of health characteristics among centenarians is important to understand if survival above age 100 is a random process or if there are patterns that drive the survival improvements of the super-select. No commonalities among health characteristics might explain the lack of survival improvements observed in the mean lifespan of centenarians [20].

The aim of the study is to reveal the health characteristics that distinguish super-selected lives surviving more than 100 years. We hypothesize that the super-select are the most resilient centenarians in terms of health, by virtue of their capacity to enhance their survival chances and reach the frontier of human survival. Robustness is therefore linked with the plasticity of ageing at the individual level, in the sense that, the most robust individuals exhibit greater malleability in their lifespans. We identify robustness via the analysis of high-quality data from the 1895, 1905 and 1910 Danish Birth Cohort Studies [34] with a statistical technique known as Latent Class Analysis [35–40]. We test the predictive power of our findings by computing the Area Under the Curve statistic (AUC, see e.g. Robin et al. [41]). The key contribution of this study is in showing a clear stratification in health and functioning among those over 100 years of age and these differences are associated with survival beyond age 100.

## Methods

Centenarian health data was retrieved from the 1895, 1905 and 1910 Danish Birth Cohort Studies. These are national population-based surveys with no exclusion criteria. All individuals born in 1895, 1905 and 1910 in Denmark were contacted to be interviewed and physically and cognitively tested during the year they would have turned 100 years. The 1895 cohort comprised of 207 out of 276 (75%) invited to participate and was examined by a geriatrician and a nurse. The assessments of the 1905 and 1910 cohorts were conducted by a specialized survey agency and comprised of 256 out of 439 (59%) and 273 out of 428 (63%) invited participants respectively. If someone was unable to participate because of their health status, a proxy respondent was invited to participate in the interview.

We use four indicators to capture different health dimensions: physical ability, functional status, cognitive status, and self-rated health. The selection of the indicators was based on previous studies showing that these characteristics are related to the survival of nonagenarians [27]. The Chair Stand test was used to assess physical ability as it has been shown to be associated with lower body strength, disability, and survival at older ages in several studies [26, 27, 30, 42–44]. Individuals who can stand up from a chair without the use of arms are in better physical health than those who need to use hands or those who cannot [42]. Functional status was assessed by five questions out of eleven questions regarding the ability to perform activities of daily living (ADL): bathing, dressing, toileting, ability to walk and feeding. These five questions were used to calculate the Katz’ disability score, where individuals were categorized into according to their answers [28, 45]. The cognitive status of centenarians was evaluated using the Mini-Mental State Examination (MMSE), which considers 19 questions. Such questions range from recalling dates and places (e.g. “What day of the week is it today?”) to those where the individual is asked to perform arithmetic calculations (e.g. “Now I will ask you to deduct 7 from 100. Then you deduct 7 from the number you arrived at and continue to deduct 7 until you are asked to stop”). The higher the MMSE score, the better the cognitive status (0–30). We divided it into three categories: 24–30 indicates no cognitive impairment, 18–23 mild cognitive impairment and 0–17 severe cognitive impairment. This categorization is based on previous studies [26, 27, 30, 46]. It is important to note that five of the MMSE questions cannot be answered by individuals who are visually impaired. However, we used the results from the completed test to impute the missing values due to being visually impaired, hereby lowering this bias. Self-rated health answers were classified into three categories: “excellent or good”, “acceptable” and “poor or very poor” [47].

It is worth noting that the questionnaire used in the assessment of health characteristics of centenarians for the 1895 cohort is slightly different from the one used for the 1905 and 1910 cohorts. First, the 1895 cohort survey does not include the Chair Stand test. Second, in the 1895 cohort, self-rated health was assessed with the question “Do you feel well considering your age?” The answers were (1) yes, (2) no and (3) reasonable. For the 1905 and 1910 cohorts, Self-Rated health was assessed with the question “All things considered, how do you consider the present status of your health?”. The answers were (1) very good, (2) good, (3) acceptable, (4) bad and (5) very bad. The answers of the 1905 and 1910 questionnaires were grouped into three categories (1) very good/good, (2) acceptable and (3) bad/very bad to match the three categories of the 1895 questionnaire. In addition, there were too few observations in the very bad and very good categories. The three-item categorization of self-rated health is also followed in previous studies of nonagenarians [26, 30, 48]. It is important to highlight that because of these differences in questionaries, results from the 1895 are not directly comparable to the other two cohorts (1905 and 1910). Detailed information about the surveys is available in [34].

The four indicators of health considered in the analysis exhibited missing values (see Supplemental Material). To handle them without introducing bias into our results, we created a “not tested” category for Chair Stand, MMSE and Self-Rated health to classify individuals that have missing values because they could not be tested due to their very poor health. For the Chair Stand score, individuals with missing values who could not perform all eleven questions regarding activities of daily living (ADL) in the survey were included in the “not tested” category. For MMSE and Self-Rated health, we categorized those individuals that reported missing values, but with the answers provided by a proxy respondent, as “not tested”. The rationale being that these tests cannot be performed by proxy respondents. For the Katz’s disability score we did not create a “not tested” category. However, this score reported very few missing values (2 individuals in each cohort). The creation of the “not tested” category allowed us to considerably reduce the number of missing values for participants who were unable to respond due to ill health [36]. However, there were still some missing values in the dataset (see Table A4 in Supplemental Material). Thus, we remove individuals who have missing values in at least one of the variables in the analysis.^2^

The date of death of each centenarian in Denmark (participants and non-participants) was retrieved from the Danish Civil Registration System. Some survey participants died before turning age 100 (e.g. ages 99.7, 99.5, etc.). We excluded these individuals from the main analysis to avoid immortal time bias in the calculation of survival probabilities [49]. After removing individuals with missing values in at least one of the variables in the analysis and those that did not survive to age 100 (37 in the 1895 cohort, 36 in the 1905 cohort and 49 in the 1910 cohort, see Supplemental Material), we analyse 170 individuals in the 1895 Cohort; 195 individuals in the 1905 Cohort and 223 in the 1910 Cohort. Tables A5 and A9 of the Supplemental Material show the characteristics of individuals included in the analysis. To test if our data is representative of the entire population, we use the log-rank test to compare survival trajectories of participants included in the analysis against those that did not participated in the survey. Survival trajectories of both groups (participants included in the analysis and non-participants) for the 1905 and 1910 cohorts are similar, which indicates that data used in our analysis is representative of national population of Danish centenarians for those cohorts. For the 1895 cohort, survival trajectories of individuals included in the analysis are statistically different from the survival trajectories of non-participants. This indicates a possible health selection in the 1895 cohort. We still analyse data of the cohort 1895 to determine if their health characteristics differ from the health characteristics of the 1905 and 1910 cohorts.

### Statistical analysis

We perform a Latent Class Analysis (LCA) to shed light on the unobserved heterogeneity in health among Danish centenarians. LCA is a statistical method used to identify unobserved classes of individuals via observed categorical variables [36–40, 50]. By considering several individual characteristics, the LCA determines individual probabilities of belonging to the latent classes and probabilities of finding a person with a certain characteristic in each class. Reference [35] provides a thorough explanation about the LCA model and in the Supplemental Material we provide more details about the specific LCA setting used in this study. Individuals in each class share similar characteristics and at the same time, they are different from individuals in other classes. Our aim is to identify health classes to further contrast the survivorship of individuals belonging to each of them. We consider different dimensions of health in the LCA: physical health (Chair Stand test), functional status (Katz’s Disability Index), cognitive impairment (MMSE) and Self-Rated Health. It is known that there are sex differences in health and survival among centenarians [51]. For this reason, we included sex as a covariate that allows us to place individuals into classes [35]. We could not stratify the analysis by sex because of the number of male centenarians that participated in the study is much smaller than the number of female centenarians in the study (see Table A5 in the Supplemental Material for details).

We performed LCA for each cohort. Since individuals in the 1895 cohort are not directly comparable to the ones in 1905 and 1910 due to differences in the questionnaire used and their survival trajectories differ from the non-participants (see details in Data section), we present the analysis of the 1895 cohort in the Supplemental Material and focus here on the 1905 and 1910 cohorts. For each cohort, various LCAs were performed by changing the number of classes in each iteration, from two to six. We considered six health classes to be the maximum possible in each cohort. More than six classes would imply high heterogeneity in health patterns but also small and meaningless classes. The optimal number of classes was selected by looking at the Akaike and Bayesian Information Criteria (AIC and BIC respectively) but also considering the health patterns and size of each class. Once the optimal number of classes in each cohort was obtained, each centenarian was assigned to a single health class. Then, based on their ages at death, we computed survival curves and the associated 95% confidence intervals by health class and by cohort using the Kaplan-Meier estimator. We assess whether there are differences in survival among the different health classes by computing the log-rank test. This test compares the entire survival experience between groups and can be thought of as a test of whether the survival curves are identical (overlapping) or not [52].

Finally, we estimated the area under the curve (AUC) to test the ability of health classes to predict the chance of surviving to the frontier of survival. The AUC ranges from 0 to 1; a higher AUC implies a better prediction [41]. We define the frontier of survival [2, 53] as the 95th percentile of the centenarian age-at-death distribution. Note that such ages change across cohorts according to mortality improvements. In Table 1 we show such ages and values for the AUC calculated for different percentiles.

## Results

Results from the Latent Class Analysis (LCA) indicate that the optimal number of health classes for the 1905 and 1910 cohorts is three (see Supplemental Material). For the 1895 cohort the optimal number of health classes is two, which indicates that there is less heterogeneity in health for this cohort possibly due to health selection. Indeed, as indicated in Section 2, survival trajectories of survey participants are statistically different to those that did not participate in the survey (see Table A1 in the Supplemental Material). Therefore, the results for the 1895 cohort are not nationally representative. In this section, we describe and compare the results of the 1905 and 1910 cohorts only (which are country representative). Results for the 1895 cohort can be found in the Supplemental Material.

Sex, included in the model as a covariate, is not statistically significant in either of the cohorts. This could be because most of centenarians are females (around 80% in each cohort). In the Supplemental Material we include a sensitivity analysis where only females are considered. The LCA health classes obtained from females-only analysis are practically the same as the ones obtained in the original analysis. This could be attributed to the fact that most of centenarians are women but also that health differences among sexes are already present in the health dimensions included in the LCA.

**Fig. 1:**
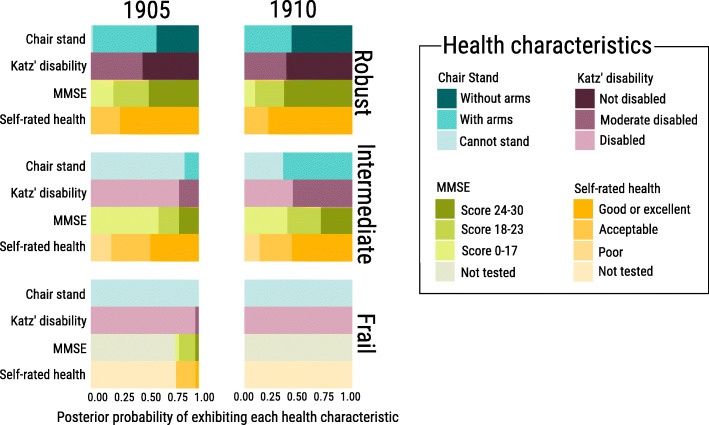
**Class membership probabilities by health class for the 1905 and 1910 cohorts, both sexes**. Note: columns represent the cohort of analysis (1905 and 1910) and rows portray the three health classes (Robust, Intermediate and Frail). Results are obtained from the Latent Class Analysis

Every LCA class is composed of individuals who share similar health characteristics. Figure 1 shows the composition of each class for the 1905 and 1910 cohorts. Based on their characteristics, we denote the classes as robust, frail and intermediate. Each bar represents a health characteristic and the size of the coloured bar depicts the probability of depicting such characteristic. For example, robust centenarians have a 44% chance of being able to stand up from a chair with the use of hands (aqua green bar) and a 56% of being able to do so without using hands (dark green bar).

Robust centenarians comprise around 117 individuals (60%) of the 1905 and 90 individuals (40%) of the 1910 cohort population. They are likely to stand up from chairs by using their arms and have high probabilities of not being physically disabled at all or being only moderately disabled. It is likely that most of them do not show significant cognitive impairment. The majority perceive their health as good. Frail centenarians on the other hand, are likely to not being able to stand up from a chair and reporting physical disability. Due to their poor health, many of them could not be tested for their cognitive status and self-rated health. Frail centenarians comprise 16% and 17% of the 1905 and 1910 cohorts respectively (around 35 individuals in each cohort). Finally, the intermediate health class comprises 24% and 42% of the 1905 and 1910 cohorts respectively. This class includes centenarians who physically and cognitively perform worse than the robust centenarians. Most of them perceive their own health to be good or acceptable.

It has been shown that nonagenarians from younger cohorts perform better in health and functioning than those from older cohorts [30]. Similar improvements in health and functioning are also portrayed in our analysis for centenarians. For example, in Fig. 1 we observe that the intermediate class of the 1910 is comprised by individuals that are more likely to be in better health in comparison to those in the intermediate class from 1905. Likewise, there is a larger share of individuals in the intermediate class in 1910 than in 1905. The robust health profile of the 1910 class is also slightly better than the 1905 class. Both health classes (robust and intermediate) comprise together around 82% of individuals in each cohort, while the frail class comprises around 18% of individuals. This indicates that improvements in health and functioning in health across cohorts are reflected in better health profiles for the robust and intermediate classes.

**Fig. 2:**
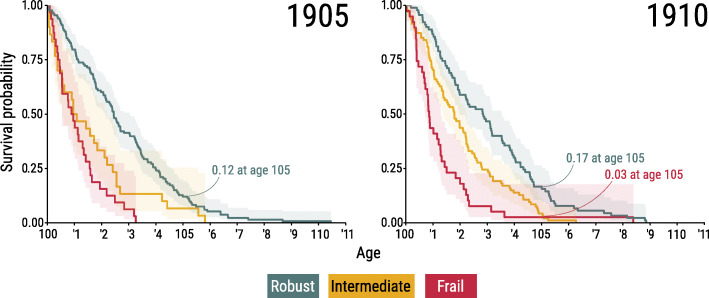
Survival probabilities above age 100 by health class and associated 95% confidence intervals for the 1905 and 1910 birth cohorts, both sexes

As mentioned above, improvements in health and functioning are reflected in higher health standards for the robust and intermediate classes in 1910. Still, the characteristics of the robust centenarians are very similar across the 1905 and 1910 cohorts (see Table A6 in Supplemental Material). Despite of not being directly comparable, the robust health class in the 1895 cohort resembles the robust health classes in the 1905 and 1910 cohorts. These commonalities in health classes across cohorts support our hypothesis about a group of centenarians outperforming in health outcomes. Thus, the question arises: are the robust centenarians also outperforming in survival? To answer this question, we computed survival curves and the associated 95% confidence intervals for the three health classes found in each cohort. Figure 2 shows the results for the 1905 and 1910 cohorts.

Figure 2 shows clear differences in survival among health classes with generally non-overlapping confidence intervals. Note, however, that at the very highest ages, the confidence bands grow wider and tend to overlap due to the very small number of survivors at those ages. Nonetheless, the log-rank test confirms formally that the three survival curves are statistically distinct (see Supplemental Material). Robust centenarians live longer than those in the other two health classes. In the 1905 cohort, their probability of survival to 105 is 0.12. For the 1910 cohort, the equivalent survival probability is 0.17, which is almost six times that for those in the frail health class. A survival gap between the robust and frail classes is also present in the 1895 cohort (see Figure A2 in Supplemental Material).

Next, we tested the ability of health classes to predict survivorship to the frontier of survival, (defined by Medford et al (2019) as the 95^th^ percentile of the centenarian age-at-death distribution) by computing the AUC (area under the curve). Depending on the percentile, AUC ranged between 0.65 and 0.68 for the 1905 cohort and 0.71 and 0.76 for the 1910 cohort (see Table 1). For the 1895 cohort, the area under the curve was estimated to be around 0.70. The AUC shows that the health class is a good predictor for reaching the frontier of survival. In particular, the AUC is consistently greater for younger cohorts, which indicates that the LCA health classes are slightly better at predicting survival of centenarians in the 1910 cohort than for centenarians in the 1905 cohort.

In a previous study, Thinggaard et al. [26] showed that the combination of Chair Stand and MMSE scores are good predictors of survival among nonagenarians so we compare the predictive ability of this approach with our LCA health classes.^3^ Both approaches (LCA health classes and Thinggaard et al. [26]) are useful in determining the survival chances to extreme ages (see Supplemental Material). However, our LCA health classes provide a more thorough description of individual health, enabling us to identify similarities in the health of centenarians. The LCA health classification provides a framework to determine the traits involved in the optimal pathways of healthy ageing.

**Table 1:** Ability of health categorization to predict survivorship to the frontier of survival measured by the area under the curve (AUC) statistic. 1905 and 1910 cohorts

	1905 Cohort		1910 Cohort	
Percentile	Age	AUC	Age	AUC
95th	105.61	0.65	105.72	0.71
96th	105.95	0.68	106.08	0.71
97th	106.26	0.68	106.39	0.76
98th	106.95	0.68	107.09	0.76
99th	107.94	0.68	108.15	0.73

Note: The AUC ranges from 0 to 1; a higher AUC implies a better prediction. Medford et al. (2019) define the frontier of survival as the 95th percentile of the centenarian age-at-death distribution. We included upper percentiles as a robustness check

### Sensitivity analysis

The focus of the present study is the relationship between health and survival trajectories of centenarians. For this reason, the LCA health classes only consider health dimensions (i.e. disability, functional health, cognitive status, and self-rated health). The selection of such health indicators is based on previous studies showing their association to survival at high advanced ages [26, 27, 30]. Nonetheless, we test how the class membership of the LCA health classes is affected when adding other factors. Specifically, we performed a sensitivity analysis of the LCA health classes by including information about smoking behaviour of the centenarians in addition to the four dimensions of health mentioned above. We show that the inclusion of smoking does not affect the identification of health classes (see Supplemental Material). This finding is in line with previous studies showing that smoking behaviour is not related to survival at high advanced ages [26, 27].

Next, we performed a sensitivity analysis where we only consider individuals with complete information (i.e. the “non tested” categories in Chair Stand, MMSE and Self Rated Health were not created, and all missing values were removed). Therefore, the sample size was reduced substantially as we only considered 170 individuals from the 1905 cohort and 182 participants from the 1910 cohort. Still, health classes remain identifiable (Figure A6 of Supplemental Material). For example, the robust class remains almost identical to the LCA analysis in Fig. 1 (i.e. when including the “no tested” category). The reason for this is that individuals with missing data are individuals in worse state of health, and they are allocated in the frail health classes. With this sensitivity analysis we confirm that there are no biases in the health classes introduced by the “not tested” category.

We performed two additional sensitivity analysis. In the first of them, we only considered females in the computation of LCA health classes. As described at the beginning of the Results section, this analysis is motivated by the fact that most centenarians in our data are females. Second, we performed a LCA by including all individuals that died before age 100. In both analyses we obtained similar results to the ones from the original LCA health classifications. Thus, we conclude that our analysis adequately captures the relationship between unobserved health categories and survival at extremely old ages. All the results from the sensitivity analyses can be found in the Supplemental Material.

## Discussion

Those surviving to the oldest ages (i.e. beyond age 105) had better health at age 100 than other survivors from their cohort. The major contributions of this study are that (i) we show the existence of a clear stratification in health and functioning among those 100 years of age and (ii) we shed light on the characteristics of the super-select centenarians (i.e. those surviving to age 105 and above). To do so, we use a high quality dataset [34] and consider different dimensions of health: physical health (Chair Stand test), functional status (Katz’s disability Index), cognitive impairment (MMSE) and Self-Rated Health which when taken together provide a well-rounded view of centenarian health and functioning.

The majority of centenarians are females and the most distinctive characteristics of the robust cluster versus the other health clusters stem from their outperformance in physical, functional and cognitive health. Most of them perceive their own health to be good or excellent. This perhaps could explain the upward trend in lifespans previously observed within this group [2]. In contrast, the intermediate and frail individuals show greater levels of physical and cognitive impairment and they have lower chances of surviving in comparison to those in the robust health class.

It was previously believed that at highest ages, the chances of survival were mostly random events [54, 55]. This school of thought suggests that survival is driven by stochastic determinants [5]. In reality, human survival is more idiosyncratic than this. We show that even at age 100 there are clear disparities in the survival prospects of centenarians based on their health profile. Furthermore, our study revealed that centenarians belonging to the robust health class are consistently in better health and survive the longer than the other centenarians. These super-select centenarians share similar health characteristics and were present in all the cohorts studied here: clearly identified in the 1905 and 1910 cohorts and slightly less clear cut in the 1895 cohort. However, we also show that there is selection in the 1895 cohort because the survival trajectories of the survey participants are statistically different than those that did not participated in the survey. Therefore, the results of the 1895 cohort should be taken with caution.

### Limitations of the study

One clear limitation of this study is that health characteristics are recorded only at age 100 but decline is likely to be rapid after then. At very old ages, health deterioration is likely to appear from one year to another [48]. Still, the data used in this analysis measures a sufficiently wide range of functioning so that it reasonably depicts an individual’s general health status [30, 34]. Likewise, it is unknown if similar findings are observed among the centenarians of other countries. In Sweden, for example, Medford et al. [2] do not find a super-select group with increased plasticity of individual lifespans. It would be interesting to determine if a robust health-class is found in Sweden and to compare the results with our findings.

We also acknowledge that some heterogeneity in survival is still uncounted in our analysis and this could be attributed to some stochastic process. An analysis with more comprehensive measures (e.g. a comprehensive geriatric assessment) of the general health of centenarians could be useful to disclose such heterogeneity. However, at present, we do not count with such data, which is a limitation of the study.

Apart from health, other factors such as socio-economic factors (i.e. education, income, etc.), lifestyle (e.g. living arrangements, calorie intake), genetic endowments and demographic characteristics might be useful to depict a broader centenarian phenotype. However, adding too many indicators to the LCA analysis might become problematic due to our small sample sizes. This could lead to meaningless LCA classes (i.e. empty classes). Instead, a similar approach as Goldman et al. [56] could be implemented to this aim.

## Conclusion

We conclude that survival advances beyond age 100 are mainly driven by this super-select group of the healthiest individuals surviving for a longer time. This is not to say that those in poor health have not been living longer as well. They have been. However, the super-select lives have been living longer than any other group and any further pushing of the frontier of survival forward will most likely be by those in the most robust health and not those in poor health. Any improvements in the dimensions of health studied here could lead to a higher prevalence of robust centenarians and ultimately to a longer living population.

## Supplementary Information

**Additional file 1: Table A1.** Survival probabilities above age 100 for participants and non-participants and associated 95% confidence intervals for the 1895 cohort.

**Additional file 2: Table A2.** Survival probabilities above age 100 for participants and non-participants and associated 95% confidence intervals for the 1905 cohort.

**Additional file 3: Table A3.** Survival probabilities above age 100 for participants and non-participants and associated 95% confidence intervals for the 1910 cohort.

**Additional file 4: Table A4.** Description of participants included in the analysis and missing values per health characteristic. Cohorts 1895, 1905 and 1910.

**Additional file 5: Table A5.** Summary statistics by gender for the cohorts 1905 and 1910.

**Additional file 6: Table A6.** Comparison of the class membership probabilities of the robust class for the 1905 and 1910 cohorts.

**Additional file 7: Table A7.** Survival probabilities above age 100 by health class and associated 95% confidence intervals for the 1905 cohort.

**Additional file 8: Table A8.** Survival probabilities above age 100 by health class and associated 95% confidence intervals for the 1910 cohort. Health classes were obtained from the Latent Class Analysis.

**Additional file 9: Table A9.** Summary statistics by gender for the cohort 1895.

**Additional file 10: Figure A1.** Class membership probabilities by health class for the 1895 cohort, both sexes.

**Additional file 11: Figure A2.** Survival probabilities above age 100 by health class and associated 95% confidence intervals for the cohort 1895, both sexes.

**Additional file 12: Table A10.** Survival probabilities above age 100 by health class and associated 95% confidence intervals for the 1895 cohort.

**Additional file 13: Table A11.** Goodness of fit for the Latent Class Analysis varying the number of clusters. Cohorts 1895, 1905, 1910.

**Additional file 14: Figure A3.** Class membership probabilities by health class for the 1905 and 1910 cohorts considering only females.

**Additional file 15: Figure A4.** Survival probabilities above age 100 by health class and associated 95% confidence intervals for the 1905 and 1910 cohorts considering only females in the Latent Class Analysis.

**Additional file 16: Table A12.** Area under the curve by percentile for the 1905 and 1910 cohorts considering only females in the analysis.

**Additional file 17: Figure A5.** Class membership probabilities by health dimension for the 1905 and 1910 cohorts including Smoking in the Latent Class Analysis, both sexes.

**Additional file 18: Figure A6.** Survival probabilities above age 100 by health class and associated 95% confidence intervals for the 1905 and 1910 cohorts including Smoking in the Latent Class Analysis, both sexes.

**Additional file 19: Table A13.** Area under the curve by percentile for the 1905 and 1910 cohorts including Smoking in the analysis.

**Additional file 20: Figure A7.** Class membership probabilities by health dimension for the 1905 and 1910 cohorts including only individuals with complete observations, without the creation of “no tested” category.

**Additional file 21: Figure A8.** Class membership probabilities by health dimension for the 1905 and 1910 cohorts including those individuals that died before age 100 in the Latent Class Analysis, both sexes.

**Additional file 22: Table A14.** Area under the curve by percentile for the 1905 and 1910 cohorts using Chair Stand (able to stand with and without hands) and MMSE (>24).

## Data Availability

Data supporting the findings of this study were used under license of the Regional Committees on Health Research Ethics for Southern Denmark (https://en.nvk.dk/). Data are available from the authors upon request.

## Abbreviations

LCA: Latent Class Analysis
BIC: Bayesian Information Criterion
AUC: Area Under the Curve
MMSE: Mini-Mental State Examination
ADL: Activities of Daily Living

## Footnotes

1 In Denmark in 2019 around 60% of female and 66% of male centenarians die by the age of 102 (Human Mortality Database, 2020).

2 The use of statistical imputation techniques like mean substitution or multiple imputation was avoided because these procedures might bias the results of the Latent Class Analysis and make comparisons among cohorts more uncertain. Therefore, we performed the analysis considering only the individuals that have complete values.

3 Following Thinggaard et al (2016) approach, we categorise those that can stand up with and without hands from their chair and having a MMSE > 24 as robust. Under this approach, the AUC ranged between 0.60 and 0.72 for the cohort 1905 and between 0.61 and 0.66 for the cohort 1910. See Table A14 in the Supplemental Material.
