# Supplementary material for: Stratification in health and survival after age 100: evidence from Danish centenarians
Source: BMC Geriatr. 2021 Jul 1;21:406. doi: 10.1186/s12877-021-02326-3 (PMC8252309; doi:10.1186/s12877-021-02326-3)
Supplement: Supplementary file 1 — Additional file 1: Table A1. Survival probabilities above age 100 for participants and non-participants and associated 95% confidence intervals for the 1895 cohort. [file 12877_2021_2326_MOESM1_ESM.docx]

Supplemental Material for

**Stratification in health and survival after age 100: Evidence from Danish centenarians**

Jesús-Adrián Alvarez^1*^, Anthony Medford^1^, Cosmo Strozza^1^, Mikael Thinggaard^2^, Kaare Christensen^2,3^

^1^Interdisciplinary Centre on Population Dynamics, University of Southern Denmark, 5000, Odense, Denmark.

^2^Danish Aging Research Centre, Epidemiology, Biostatistics and Biodemography, Department of Public Health, University of Southern Denmark, Odense C.

^3^Department of Clinical Genetics and Department of Clinical Biochemistry and Pharmacology, Odense University Hospital, Denmark.

*Corresponding author. Email: [alvarez@sdu.dk](mailto:alvarez@sdu.dk)

1. **Comparison of survival trajectories of participants of Danish Birth Cohort Studies vs non-participants for the 1895, 1905 and 1910 cohorts**

**Table A1. Survival probabilities above age 100 for participants and non-participants**

**and associated 95% confidence intervals for the 1895 cohort.**

|  | ***Participants*** | |  | ***No Participants*** | |
| --- | --- | --- | --- | --- | --- |
| ***Age*** | ***Survival probability*** | ***CI (95%)*** |  | ***Survival probability*** | ***CI (95%)*** |
| ***100.0*** | 1.00 | (1,1) |  | 1.00 | (1,1) |
| ***100.5*** | 0.83 | (0.78,0.88) |  | 0.69 | (0.59,0.81) |
| ***101.0*** | 0.64 | (0.58,0.71) |  | 0.47 | (0.37,0.61) |
| ***101.5*** | 0.48 | (0.42,0.56) |  | 0.30 | (0.21,0.44) |
| ***102.0*** | 0.35 | (0.29,0.42) |  | 0.24 | (0.16,0.37) |
| ***102.5*** | 0.29 | (0.24,0.36) |  | 0.16 | (0.09,0.27) |
| ***103.0*** | 0.20 | (0.15,0.26) |  | 0.13 | (0.07,0.24) |
| ***103.5*** | 0.15 | (0.11,0.21) |  | 0.07 | (0.03,0.17) |
| ***104.0*** | 0.11 | (0.08,0.16) |  | 0.05 | (0.02,0.15) |
| ***104.5*** | 0.09 | (0.06,0.14) |  | 0.04 | (0.01,0.13) |
| ***105.0*** | 0.08 | (0.05,0.13) |  | 0.01 | (0,0.1) |
| ***105.5*** | 0.06 | (0.03,0.1) |  | 0.01 | (0,0.1) |
| ***106.0*** | 0.04 | (0.02,0.08) |  | 0.01 | (0,0.1) |
| ***106.5*** | 0.04 | (0.02,0.08) |  |  |  |
| ***107.0*** | 0.03 | (0.01,0.06) |  |  |  |
| ***107.5*** | 0.02 | (0.01,0.06) |  |  |  |
| ***108.0*** | 0.01 | (0,0.04) |  |  |  |
| ***108.5*** | 0.00 | (0,0.03) |  |  |  |
| ***109.0*** |  |  |  |  |  |
| ***109.5*** |  |  |  |  |  |
| ***110.0*** |  |  |  |  |  |

Log-rank test p-value=0.007.
