# Supplementary material for: Stratification in health and survival after age 100: evidence from Danish centenarians
Source: BMC Geriatr. 2021 Jul 1;21:406. doi: 10.1186/s12877-021-02326-3 (PMC8252309; doi:10.1186/s12877-021-02326-3)
Supplement: Supplementary file 2 — Additional file 2: Table A2. Survival probabilities above age 100 for participants and non-participants and associated 95% confidence intervals for the 1905 cohort. [file 12877_2021_2326_MOESM2_ESM.docx]

**Table A2. Survival probabilities above age 100 for participants and non-participants**

**and associated 95% confidence intervals for the 1905 cohort.**

|  | ***Participants*** | |  | ***No Participants*** | |
| --- | --- | --- | --- | --- | --- |
| ***Age*** | ***Survival probability*** | ***CI (95%)*** |  | ***Survival probability*** | ***CI (95%)*** |
| ***100.0*** | 1.00 | (1,1) |  | 1.00 | (1,1) |
| ***100.5*** | 0.82 | (0.77,0.87) |  | 0.77 | (0.7,0.84) |
| ***101.0*** | 0.66 | (0.61,0.73) |  | 0.59 | (0.52,0.67) |
| ***101.5*** | 0.56 | (0.5,0.63) |  | 0.43 | (0.36,0.52) |
| ***102.0*** | 0.45 | (0.39,0.52) |  | 0.37 | (0.29,0.45) |
| ***102.5*** | 0.34 | (0.28,0.4) |  | 0.30 | (0.23,0.38) |
| ***103.0*** | 0.28 | (0.23,0.35) |  | 0.23 | (0.17,0.31) |
| ***103.5*** | 0.20 | (0.16,0.26) |  | 0.16 | (0.11,0.23) |
| ***104.0*** | 0.16 | (0.13,0.22) |  | 0.12 | (0.07,0.18) |
| ***104.5*** | 0.11 | (0.08,0.17) |  | 0.10 | (0.06,0.17) |
| ***105.0*** | 0.07 | (0.05,0.12) |  | 0.07 | (0.03,0.13) |
| ***105.5*** | 0.05 | (0.03,0.09) |  | 0.04 | (0.01,0.09) |
| ***106.0*** | 0.03 | (0.01,0.06) |  | 0.03 | (0.01,0.07) |
| ***106.5*** | 0.02 | (0.01,5) |  | 0.01 | (0,0.06) |
| ***107.0*** | 0.01 | (0,0.04) |  | 0.01 | (0,0.05) |
| ***107.5*** | 0.01 | (0,0.03) |  | 0.01 | (0,0.05) |
| ***108.0*** | 0.01 | (0,0.03) |  | 0.01 | (0,0.05) |
| ***108.5*** | 0.01 | (0,0.03) |  |  |  |
| ***109.0*** | 0.01 | (0,0.03) |  |  |  |
| ***109.5*** | 0.01 | (0,0.03) |  |  |  |
| ***110.0*** | 0.01 | (0,0.03) |  |  |  |

Log-rank test p-value =0.13
