# Supplementary material for: Stratification in health and survival after age 100: evidence from Danish centenarians
Source: BMC Geriatr. 2021 Jul 1;21:406. doi: 10.1186/s12877-021-02326-3 (PMC8252309; doi:10.1186/s12877-021-02326-3)
Supplement: Supplementary file 3 — Additional file 3: Table A3. Survival probabilities above age 100 for participants and non-participants and associated 95% confidence intervals for the 1910 cohort. [file 12877_2021_2326_MOESM3_ESM.docx]

**Table A3. Survival probabilities above age 100 for participants and non-participants**

**and associated 95% confidence intervals for the 1910 cohort.**

|  | ***Participants*** | |  | ***No Participants*** | |
| --- | --- | --- | --- | --- | --- |
| ***Age*** | ***Survival probability*** | ***CI (95%)*** |  | ***Survival probability*** | ***CI (95%)*** |
| ***100.0*** | 1.00 | (1,1) |  | 1.00 | (1,1) |
| ***100.5*** | 0.88 | (0.08,0.92) |  | 0.81 | (0.75,0.88) |
| ***101.0*** | 0.72 | (0.66,0.77) |  | 0.64 | (0.56,0.72) |
| ***101.5*** | 0.55 | (0.49,0.62) |  | 0.46 | (0.38,0.55) |
| ***102.0*** | 0.41 | (0.35,0.47) |  | 0.35 | (0.28,0.44) |
| ***102.5*** | 0.32 | (0.27,0.38) |  | 0.26 | (0.19,0.34) |
| ***103.0*** | 0.27 | (0.22,33) |  | 0.19 | (0.13,0.26) |
| ***103.5*** | 0.22 | (0.18,0.27) |  | 0.11 | (0.07,0.18) |
| ***104.0*** | 0.27 | (0.12,0.21) |  | 0.09 | (0.06,0.16) |
| ***104.5*** | 0.13 | (0.09,0.17) |  | 0.08 | (0.05,0.14) |
| ***105.0*** | 0.08 | (0.05,0.12) |  | 0.06 | (0.03,0.12) |
| ***105.5*** | 0.04 | (0.02,0.07) |  | 0.06 | (0.03,0.11) |
| ***106.0*** | 0.04 | (0.02,0.07) |  | 0.04 | (0.02,0.09) |
| ***106.5*** | 0.03 | (0.01,0.05) |  | 0.03 | (0.02,0.08) |
| ***107.0*** | 0.03 | (0.01,0.05) |  | 0.03 | (0.01,0.07) |
| ***107.5*** | 0.02 | (0.01,0.05) |  | 0.02 | (0.01,0.06) |
| ***108.0*** | 0.01 | (0,0.04) |  | 0.01 | (0.01,0.06) |
| ***108.5*** | 0.01 | (0,0.03) |  | 0.01 | (0,0.05) |
| ***109.0*** | 0.01 | (0,0.03) |  |  |  |
| ***109.5*** | 0.01 | (0,0.03) |  |  |  |
| ***110.0*** | 0.01 | (0,0.03) |  |  |  |

Log-rank test p-value =0.08

According to the p-value of the log-rank tests, the survival trajectories of the participants of the 1905 and 1910 are not statistically different. However, for the 1895 the p-value of the log-rank test indicates that there are differences between the survival trajectories of the participants of the study and non-participants. This indicates that the data for the 1895 cohort is not country representative. The survival advantage for the participants could be attributed to health selection.
