# Supplementary material for: Stratification in health and survival after age 100: evidence from Danish centenarians
Source: BMC Geriatr. 2021 Jul 1;21:406. doi: 10.1186/s12877-021-02326-3 (PMC8252309; doi:10.1186/s12877-021-02326-3)
Supplement: Supplementary file 4 — Additional file 4: Table A4. Description of participants included in the analysis and missing values per health characteristic. Cohorts 1895, 1905 and 1910. [file 12877_2021_2326_MOESM4_ESM.docx]

**Table A4. Description of participants included in the analysis and missing values per health characteristic. Cohorts 1895, 1905 and 1910.**

|  |  | **Cohort 1895** | **Cohort 1905** | **Cohort 1910** |
| --- | --- | --- | --- | --- |
| **Total participants** | | **207** | **256** | **273** |
|  | Participants that died before turning age 100 | 0 | -25 | -1 |
|  | Participants in the study after removing those that did not turn age 100 | 207 | 231 | 272 |
|  |  |  |  |  |
|  | **Missing values*** |  |  |  |
|  | MMSE (%) | 64 (31) | 56 (24) | 84 (31) |
|  | Chair Stand (%) | NA | 56 (24) | 82 (30) |
|  | Self-Rated Health (%) | 30 (14) | 51 (22) | 76 (28) |
|  | Katz's disability index (%) | 0 | 2 (1) | 2 (1) |
|  |  |  |  |  |
|  | **Proxy respondent** | 30 | 51 | 76 |
|  | **Individuals with missing values in at least one category after the creation of the "no tested" category** | 37 | 36 | 49 |
| **Participants included in the analysis** | | **170** | **195** | **223** |
| * There are individuals that exhibit missing values in more than one category. | | | | |
| For example, for the 1905 cohort we excluded 36 individuals after creating | | | |  |
| the “no tested” category. 5 of those 36 had missing values in MMSE | | | |  |
| and 31 of the 36 had missing values in chair stand. | | |  |  |
| 2 individuals with missing values in the disability index | | |  |  |
| also have missing values in chair stand. | |  |  |  |
