# Supplementary material for: Stratification in health and survival after age 100: evidence from Danish centenarians
Source: BMC Geriatr. 2021 Jul 1;21:406. doi: 10.1186/s12877-021-02326-3 (PMC8252309; doi:10.1186/s12877-021-02326-3)
Supplement: Supplementary file 5 — Additional file 5: Table A5. Summary statistics by gender for the cohorts 1905 and 1910. [file 12877_2021_2326_MOESM5_ESM.docx]

1. **Summary statistics and survival probabilities for participants of the surveys of the 1905 and 1910 cohorts**

**Table A5. Summary statistics by gender for the cohorts 1905 and 1910.**

|  | **Males** |  |  |  | **Females** |  |  |  | **Sex differences** | |
| --- | --- | --- | --- | --- | --- | --- | --- | --- | --- | --- |
|  | **1905 cohort** | **1910 cohort** | **p-value*** |  | **1905 cohort** | **1910 cohort** | **p-value*** |  | **1905 cohort** | **1910 cohort** |
| **Participants included in the analysis, n** | 33 | 51 |  |  | 162 | 172 |  |  |  |  |
| **Mean age of interview (SD)** | 99.8 (0.3) | 100.3 (0.4) | <0.001 |  | 99.8 (0.28) | 100.35 (0.3) | <0.001 |  | 0.410 | 0.532 |
| **Mean age at death (SD)** | 102.4 (1.8) | 102.0 (1.6) | 0.294 |  | 102.3 (1.9) | 102.4 (1.9) | 0.506 |  | 0.775 | 0.142 |
| **Proxy, n (%)** |  |  | 1.000 |  |  |  | 0.117 |  | 1.000 | 0.519 |
| Yes | 4 (12.1) | 7 (13.7) |  |  | 20 (12.3) | 32 (18.6) |  |  |  |  |
| No | 29 (87.9) | 44 (86.3) |  |  | 142 (87.7) | 140 (81.1) |  |  |  |  |
| **MMSE, n (%)** |  |  | 0.957 |  |  |  | 0.244 |  | 0.284 | 0.341 |
| Score 0-17 | 5 (15.2) | 9 (17.6) |  |  | 44 (27.2) | 38 (22.1) |  |  |  |  |
| Score 18-23 | 8 (24.2) | 10 (19.6) |  |  | 47 (29.0) | 43 (25.0) |  |  |  |  |
| Score 24-30 | 16 (48.5) | 25 (49.0) |  |  | 51 (31.5) | 59 (34.3) |  |  |  |  |
| No tested | 4 (12.1) | 7 (13.7) |  |  | 20 (12.3) | 32 (18.6) |  |  |  |  |
| **Self-rated health, n (%)** |  |  | 0.647 |  |  |  | 0.177 |  | 0.683 | 0.536 |
| No tested | 4 (12.1) | 7 (13.7) |  |  | 20 (12.3) | 32 (18.6) |  |  |  |  |
| Poor | 1 (3.0) | 3 (5.9) |  |  | 13 (8.0) | 10 (15.8) |  |  |  |  |
| Acceptable | 8 (24.2) | 14 (27.5) |  |  | 45 (27.8) | 34 (19.8) |  |  |  |  |
| Good/Excellent | 20 (60.6 | 27 (51.9) |  |  | 87 (51.9) | 96 (55.8) |  |  |  |  |
| **Chair Stand, n (%)** |  |  | 0.705 |  |  |  | 0.531 |  | 0.664 | 0.641 |
| Cannot stand | 10 (30.3) | 14 (27.5) |  |  | 50 (30.9) | 55 (32.0) |  |  |  |  |
| With use of arms | 16 (48.5) | 22 (43.1) |  |  | 67 (41.4) | 78 (45.3) |  |  |  |  |
| Without use of arms | 7 (21.2) | 15 (29.4) |  |  | 45 (27.8) | 39 (22.7) |  |  |  |  |
| **Katz's disability index, n (%)** |  |  | 0.785 |  |  |  | 0.054 |  | 0.489 | 0.058 |
| Disabled | 13 (39.4) | 17 (33.3) |  |  | 49 (30.2) | 64 (37.2) |  |  |  |  |
| Moderately | 10 (30.3) | 15 (29.4) |  |  | 61 (37.7) | 72 (41.9) |  |  |  |  |
| Not disabled | 10 (30.3) | 19 (37.3) |  |  | 52 (32.1) | 36 (20.9) |  |  |  |  |
|  |  |  |  |  |  |  |  |  |  |  |
| *Test to determine equal means between the populations analysed | | | | |  |  |  |  |  |  |
| MMSE: Mini-Mental State Examination | | |  |  |  |  |  |  |  |  |
| Note: We only included individuals that do not present missing values in any of the characteristics observed. | | | | | | | |  |  |  |
