# Supplementary material for: Stratification in health and survival after age 100: evidence from Danish centenarians
Source: BMC Geriatr. 2021 Jul 1;21:406. doi: 10.1186/s12877-021-02326-3 (PMC8252309; doi:10.1186/s12877-021-02326-3)
Supplement: Supplementary file 6 — Additional file 6: Table A6. Comparison of the class membership probabilities of the robust class for the 1905 and 1910 cohorts. [file 12877_2021_2326_MOESM6_ESM.docx]

**Table A6. Comparison of the class membership probabilities of the robust class for the 1905 and 1910 cohorts.**

|  |  | **Cohort** | |
| --- | --- | --- | --- |
| **Dimension** | **Score** | **1905** | **1910** |
| **Mini-mental state examination** | 24-30 | 0.46 | 0.63 |
|  | 18-23 | 0.33 | 0.27 |
|  | 0-17 | 0.21 | 0.1 |
|  | Not tested | 0 | 0 |
|  |  |  |  |
| **Self-rated health** | Good or excellent | 0.73 | 0.78 |
|  | Acceptable | 0.27 | 0.22 |
|  | Poor | 0 | 0 |
|  | Not Tested | 0 | 0 |
|  |  |  |  |
| **Chair stand test** | Without arms | 0.39 | 0.56 |
|  | With arms | 0.59 | 0.44 |
|  | Cannot stand | 0.02 | 0 |
|  |  |  |  |
| **Katz's disability score** | Not disabled | 0.52 | 0.61 |
|  | Moderate disabled | 0.48 | 0.39 |
|  | Disabled | 0 | 0 |
|  |  |  |  |
