# Supplementary material for: Stratification in health and survival after age 100: evidence from Danish centenarians
Source: BMC Geriatr. 2021 Jul 1;21:406. doi: 10.1186/s12877-021-02326-3 (PMC8252309; doi:10.1186/s12877-021-02326-3)
Supplement: Supplementary file 7 — Additional file 7: Table A7. Survival probabilities above age 100 by health class and associated 95% confidence intervals for the 1905 cohort. [file 12877_2021_2326_MOESM7_ESM.docx]

**Table A7. Survival probabilities above age 100 by health class and associated 95% confidence intervals for the 1905 cohort.**

|  | **Robust** | |  | **Intermediate** | |  | **Frail** | |
| --- | --- | --- | --- | --- | --- | --- | --- | --- |
| **Age** | **Survival probability** | **CI (95%)** |  | **Survival probability** | **CI (95%)** |  | **Survival probability** | **CI (95%)** |
| **100.0** | 1.00 | (1,1) |  | 1.00 | (1,1) |  | 1.00 | (1,1) |
| **100.5** | 0.92 | (0.87,0.97) |  | 0.70 | (0.55,0.89) |  | 0.69 | (0.54,0.87) |
| **101.0** | 0.80 | (0.73,0.87) |  | 0.50 | (0.35,0.72) |  | 0.47 | (0.32,0.68) |
| **101.5** | 0.71 | (0.64,0.8) |  | 0.43 | (0.29,0.65) |  | 0.28 | (0.16,0.49) |
| **102.0** | 0.60 | (0.52,0.69) |  | 0.33 | (0.2,0.55) |  | 0.16 | (0.07,0.35) |
| **102.5** | 0.48 | (0.4,0.57) |  | 0.23 | (0.12,0.45) |  | 0.09 | (0.03,0.28) |
| **103.0** | 0.41 | (0.34,0.51) |  | 0.13 | (0.05,0.33) |  | 0.06 | (0.02,0.24) |
| **103.5** | 0.31 | (0.24,0.4) |  | 0.13 | (0.05,0.33) |  |  |  |
| **104.0** | 0.25 | (0.18,0.33) |  | 0.13 | (0.05,0.33) |  |  |  |
| **104.5** | 0.18 | (0.13,0.26) |  | 0.07 | (0.02,0.25) |  |  |  |
| **105.0** | 0.12 | (0.08,0.19) |  | 0.07 | (0.02,0.25) |  |  |  |
| **105.5** | 0.08 | (0.04,0.14) |  | 0.07 | (0.02,0.25) |  |  |  |
| **106.0** | 0.05 | (0.03,0.11) |  |  |  |  |  |  |
| **106.5** | 0.04 | (0.02,0.09) |  |  |  |  |  |  |
| **107.0** | 0.02 | (0.01,0.07) |  |  |  |  |  |  |
| **107.5** | 0.02 | (0,0.06) |  |  |  |  |  |  |
| **108.0** | 0.02 | (0,0.06) |  |  |  |  |  |  |
| **108.5** | 0.02 | (0,0.06) |  |  |  |  |  |  |
| **109.0** | 0.01 | (0,0.05) |  |  |  |  |  |  |
| **109.5** | 0.01 | (0,0.05) |  |  |  |  |  |  |
| **110.0** | 0.01 | (0,0.05) |  |  |  |  |  |  |

Log-rank test p-value<0.001

This p-value indicates that the null hypothesis should be rejected, thus the survival curves are statistically different from each other.
