# Supplementary material for: Stratification in health and survival after age 100: evidence from Danish centenarians
Source: BMC Geriatr. 2021 Jul 1;21:406. doi: 10.1186/s12877-021-02326-3 (PMC8252309; doi:10.1186/s12877-021-02326-3)
Supplement: Supplementary file 8 — Additional file 8: Table A8. Survival probabilities above age 100 by health class and associated 95% confidence intervals for the 1910 cohort. Health classes were obtained from the Latent Class Analysis. [file 12877_2021_2326_MOESM8_ESM.docx]

**Table A8. Survival probabilities above age 100 by health class and associated 95% confidence intervals for the 1910 cohort. Health classes were obtained from the Latent Class Analysis.**

|  | **Robust** | |  | **Intermediate** | |  | **Frail** | |
| --- | --- | --- | --- | --- | --- | --- | --- | --- |
| **Age** | **Survival probability** | **CI (95%)** |  | **Survival probability** | **CI (95%)** |  | **Survival probability** | **CI (95%)** |
| **100.0** | 1.00 | (1,1) |  | 1.00 | (1,1) |  | 1.00 | (1,1) |
| **100.5** | 0.96 | (0.91,1) |  | 0.72 | (0.59,0.87) |  | 0.87 | (0.81,0.94) |
| **101.0** | 0.87 | (0.8,0.94) |  | 0.44 | (0.31,0.62) |  | 0.70 | (0.62,0.8) |
| **101.5** | 0.72 | (0.64,0.82) |  | 0.26 | (0.15,0.44) |  | 0.56 | (0.47,0.67) |
| **102.0** | 0.59 | (0.5,0.7) |  | 0.18 | (0.09,0.35) |  | 0.44 | (0.35,0.55) |
| **102.5** | 0.53 | (0.44,0.65) |  | 0.08 | (0.03,0.23) |  | 0.32 | (0.24,0.43) |
| **103.0** | 0.47 | (0.37,0.58) |  | 0.08 | (0.03,0.23) |  | 0.24 | (0.17,0.35) |
| **103.5** | 0.40 | (0.31,0.52) |  | 0.05 | (0.01,0.2) |  | 0.19 | (0.13,0.29) |
| **104.0** | 0.30 | (0.22,0.41) |  | 0.03 | (0,0.18) |  | 0.14 | (0.08,0.23) |
| **104.5** | 0.23 | (0.16,0.34) |  | 0.03 | (0,0.18) |  | 0.11 | (0.06,0.19) |
| **105.0** | 0.17 | (0.11,0.26) |  | 0.03 | (0,0.18) |  | 0.04 | (0.02,0.11) |
| **105.5** | 0.09 | (0.05,0.17) |  | 0.03 | (0,0.18) |  | 0.01 | (0,0.07) |
| **106.0** | 0.08 | (0.04,0.16) |  | 0.03 | (0,0.18) |  | 0.01 | (0,0.07) |
| **106.5** | 0.06 | (0.02,0.13) |  | 0.03 | (0,0.18) |  |  |  |
| **107.0** | 0.06 | (0.02,0.13) |  | 0.03 | (0,0.18) |  |  |  |
| **107.5** | 0.04 | (0.02,0.12) |  | 0.03 | (0,0.18) |  |  |  |
| **108.0** | 0.03 | (0.01,0.1) |  | 0.03 | (0,0.18) |  |  |  |
| **108.5** | 0.02 | (0.01,0.09) |  |  |  |  |  |  |
| **109.0** |  |  |  |  |  |  |  |  |
| **109.5** |  |  |  |  |  |  |  |  |
| **110.0** |  |  |  |  |  |  |  |  |

Log-rank test p-value <0.001

This p-value indicates that the null hypothesis should be rejected, thus the survival curves are statistically different from each other.
