# Supplementary material for: Stratification in health and survival after age 100: evidence from Danish centenarians
Source: BMC Geriatr. 2021 Jul 1;21:406. doi: 10.1186/s12877-021-02326-3 (PMC8252309; doi:10.1186/s12877-021-02326-3)
Supplement: Supplementary file 9 — Additional file 9: Table A9. Summary statistics by gender for the cohort 1895. [file 12877_2021_2326_MOESM9_ESM.docx]

1. **Analysis of the 1895 cohort**

**Table A9. Summary statistics by gender for the cohort 1895.**

|  | **1895 cohort** | | |
| --- | --- | --- | --- |
|  | **Males** | **Females** | **p-value*** |
| **Participants included in the analysis, n** | 40 | 130 |  |
| **Mean age of interview (SD)** | 100.1 (0.04) | 100.1 (0.06) | <0.001 |
| **Age of death** | 2.2 (1.58) | 2.1 (1.87) | 0.603 |
| **Proxy, n (%)** |  |  | 0.624 |
| Yes | 6 (15.0) | 24 (18.5) |  |
| No | 34 (85.0) | 106 (81.5) |  |
| **MMSE, n (%)** |  |  | 0.742 |
| Score 0-17 | 9 (22.5) | 36 (27.7) |  |
| Score 18-23 | 14 (35.0) | 38 (29.2) |  |
| Score 24-30 | 11 (27.5) | 32 (24.6) |  |
| No tested | 6 (15.0) | 24 (18.5) |  |
| **Self-rated health , n (%)** |  |  | 0.183 |
| No tested | 3 (7.5) | 20 (15.4) |  |
| Poor | 1 (2.5) | 14 (10.8) |  |
| Acceptable | 6 (15.0) | 15 (11.5) |  |
| Good/Excellent | 30 (15.0) | 81 (61.3) |  |
| **Katz's disability index, n (%)** |  |  | <0.001 |
| Disabled | 9 (22.5) | 55 (42.3) |  |
| Moderately | 19 (47.5) | 60 (46.2) |  |
| Not disabled | 12 (30.0) | 15 (11.5) |  |
| *Test to determine equal means between the populations analysed | | | |
| MMSE: Mini-Mental State Examination | |  |  |
| Note: We only included individuals that do not present | | |  |
| missing values in any of the characteristics observed. | | |  |
