# Supplementary figures and images for: Stratification in health and survival after age 100: evidence from Danish centenarians
Source: BMC Geriatr. 2021 Jul 1;21:406. doi: 10.1186/s12877-021-02326-3 (PMC8252309; doi:10.1186/s12877-021-02326-3)

**Figure A1. Class membership probabilities by health class for the 1895 cohort, both sexes.**

**
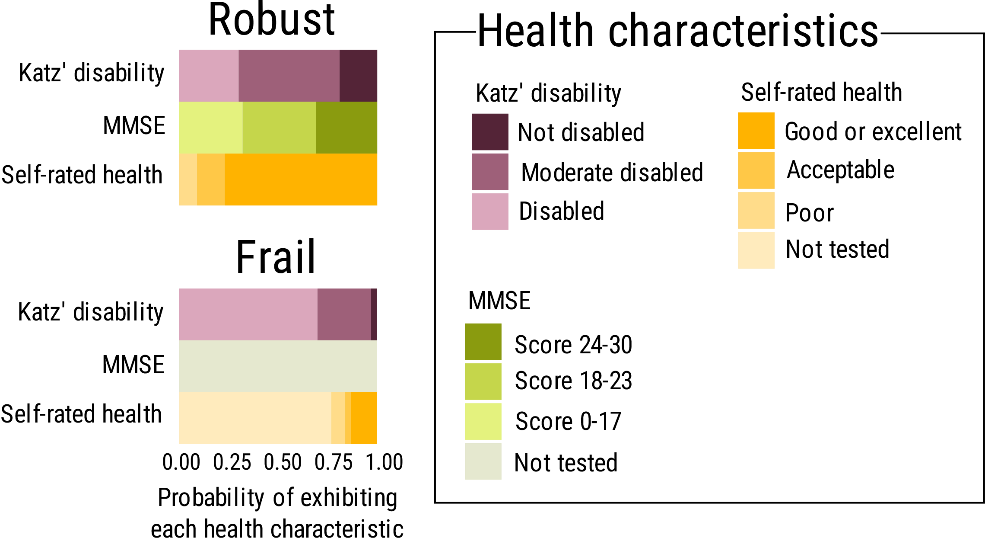
**

Supplement: Supplementary file 10 — Additional file 10: Figure A1. Class membership probabilities by health class for the 1895 cohort, both sexes. [file 12877_2021_2326_MOESM10_ESM.docx]
