# Supplementary material for: Stratification in health and survival after age 100: evidence from Danish centenarians
Source: BMC Geriatr. 2021 Jul 1;21:406. doi: 10.1186/s12877-021-02326-3 (PMC8252309; doi:10.1186/s12877-021-02326-3)
Supplement: Supplementary file 11 — Additional file 11: Figure A2. Survival probabilities above age 100 by health class and associated 95% confidence intervals for the cohort 1895, both sexes. [file 12877_2021_2326_MOESM11_ESM.docx]

**Figure A2. Survival probabilities above age 100 by health class and associated 95% confidence intervals for the cohort 1895, both sexes.**

**
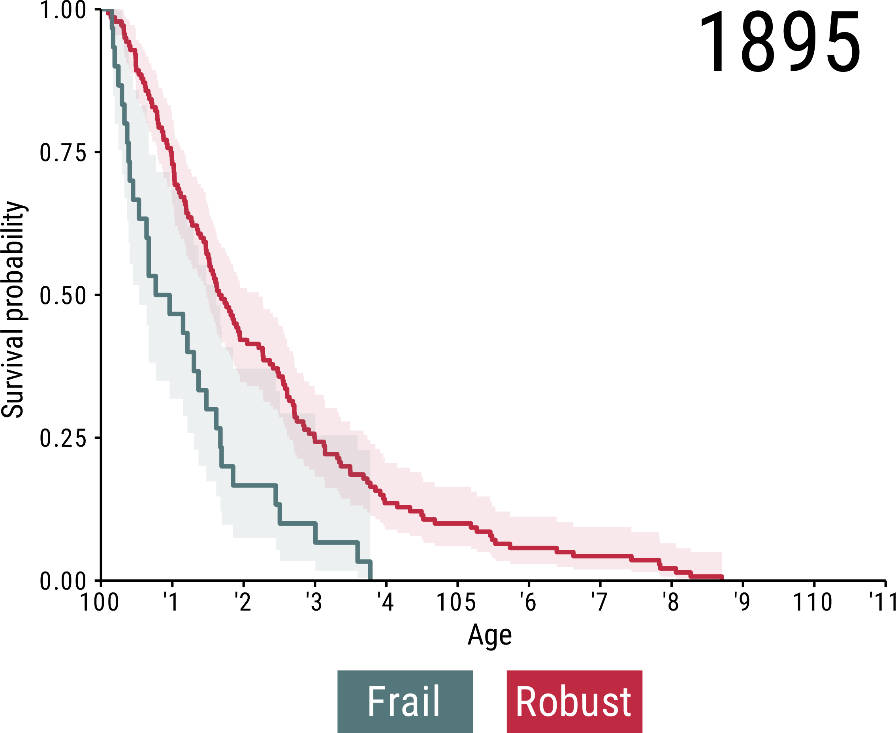
**
