# Supplementary material for: Stratification in health and survival after age 100: evidence from Danish centenarians
Source: BMC Geriatr. 2021 Jul 1;21:406. doi: 10.1186/s12877-021-02326-3 (PMC8252309; doi:10.1186/s12877-021-02326-3)
Supplement: Supplementary file 12 — Additional file 12: Table A10. Survival probabilities above age 100 by health class and associated 95% confidence intervals for the 1895 cohort. [file 12877_2021_2326_MOESM12_ESM.docx]

**Table A10. Survival probabilities above age 100 by health class and associated 95% confidence intervals for the 1895 cohort.**

|  | **Robust** | |  | **Frail** | |
| --- | --- | --- | --- | --- | --- |
| **Age** | **Survival probability** | **CI (95%)** |  | **Survival probability** | **CI (95%)** |
| **100.0** | 1.00 | (1,1) |  | 1.00 | (1,1) |
| **100.5** | 0.89 | (0.84,0.95) |  | 0.67 | (0.52,0.86) |
| **101.0** | 0.73 | (0.66,0.81) |  | 0.47 | (0.32,0.68) |
| **101.5** | 0.57 | (0.50,0.66) |  | 0.30 | (0.17,0.52) |
| **102.0** | 0.42 | (0.35,0.51) |  | 0.17 | (0.07,0.37) |
| **102.5** | 0.36 | (0.29,0.45) |  | 0.13 | (0.05,0.33) |
| **103.0** | 0.24 | (0.18,0.33) |  | 0.10 | (0.03,0.29) |
| **103.5** | 0.19 | (0.13,0.26) |  | 0.07 | (0.02,0.25) |
| **104.0** | 0.14 | (0.09,0.21) |  |  |  |
| **104.5** | 0.11 | (0.07,0.18) |  |  |  |
| **105.0** | 0.10 | (0.06,0.16) |  |  |  |
| **105.5** | 0.07 | (0.04,0.13) |  |  |  |
| **106.0** | 0.06 | (0.03,0.11) |  |  |  |
| **106.5** | 0.05 | (0.02,0.1) |  |  |  |
| **107.0** | 0.04 | (0.02,0.09) |  |  |  |
| **107.5** | 0.04 | (0.02,0.08) |  |  |  |
| **108.0** | 0.02 | (0.01,0.07) |  |  |  |
| **108.5** | 0.01 | (0,0.05) |  |  |  |
| **109.0** |  |  |  |  |  |
| **109.5** |  |  |  |  |  |
| **110.0** |  |  |  |  |  |

Log-rank test p-value<0.001

This p-value indicates that the null hypothesis should be rejected, which indicates that the survival curves are statistically different from each other.
