# Supplementary material for: Stratification in health and survival after age 100: evidence from Danish centenarians
Source: BMC Geriatr. 2021 Jul 1;21:406. doi: 10.1186/s12877-021-02326-3 (PMC8252309; doi:10.1186/s12877-021-02326-3)
Supplement: Supplementary file 13 — Additional file 13: Table A11. Goodness of fit for the Latent Class Analysis varying the number of clusters. Cohorts 1895, 1905, 1910. [file 12877_2021_2326_MOESM13_ESM.docx]

1. **Latent Class Analysis (LCA) model details**

Suppose a latent class model with *C* classes to be estimated with *M* categorical variables and a covariate *x*. Let $Y_{i}=\left( Y_{i1},\ldots,Y_{\mathrm{iM}} \right)$ be the vector of individual's response to the *M* variables where $Y_{im}=1,2,\ldots,r_{m}$ indicates the individual characteristic for a certain variable. Let $c_{i}=1,2,\ldots,C$ be the latent class membership of the individual to the class; let $I\left( y_{im}=k \right)$ be the indicator function that is 1 if the response of individual i to the variable m^th^ variable y is equal to *k* and 0 otherwise; let $\lambda_{c}$ be the probability of membership in each latent class and let $\rho_{mk|c}$ be the item-response probability (variable m, category k) conditional on the latent class membership (c). The latent class model is expressed as follows:

$$P\left( Y=y | x_{i} \right)=\sum_{c=1}^{C} \lambda_{c}\left( x_{i} \right)\prod_{m=1}^{M} \prod_{k=1}^{r_{m}} \rho_{mk|c} I\left( y_{im}=k \right)$$

where $\lambda_{c}\left( x_{i} \right)=P\left( C_{i}=c | x_{i} \right)$ is a standard baseline-category for the multinomial logistic model. In the case of one covariate, $\lambda$ is expressed as follows:

$$\lambda_{c}\left( x_{i} \right)=P\left( C_{i}=c | x_{i} \right)=\frac{\exp\{\beta_{0c}+x_{i}\beta_{1c}\}}{1+\sum_{j=1}^{C} \exp\{\beta_{0j}+x_{i}\beta_{1j}\}}$$

for $c = 1,\ldots,C-1$, where *C* is the reference class in the logistic regression. Finally, the log-odds of an individual falling into latent class *c* relative to the reference class *C*, giving $x_{i}$ as the value for the covariate, is:

$$\log\left( \frac{\lambda_{c|c}(x_{i})}{\lambda_{C|c}(x_{i})} \right)= \beta_{0c|c}+\beta_{1c|c}x_{i}$$

**Table A11. Goodness of fit for the Latent Class Analysis varying the number of clusters. Cohorts 1895, 1905, 1910.**

|  | **Akaike information criterion** | | |  | **Bayesian information criterion** | | |
| --- | --- | --- | --- | --- | --- | --- | --- |
| **Number of clusters** | **1895 cohort** | **1905 cohort** | **1910 cohort** |  | **1895 cohort** | **1905 cohort** | **1910 cohort** |
| 2 | 1,072.38 | 1,561.93 | 1,680.75 |  | 1,128.83 | 1,652.72 | 1,755.71 |
| 3 | 1,176.21 | 1,541.43 | 1,601.32 |  | 1,264.01 | 1,633.94 | 1,717.17 |
| 4 | 1,068.78 | 1,640.55 | 1,655.70 |  | 1,187.94 | 1,791.11 | 1,812.43 |
| 5 | 1,163.47 | 1,899.22 | 2,003.25 |  | 1,313.99 | 2,089.05 | 2,200.87 |
| 6 | 1,183.47 | 1,942.05 | 1,761.15 |  | 1,365.35 | 2,171.16 | 1,999.65 |
