# Supplementary material for: Stratification in health and survival after age 100: evidence from Danish centenarians
Source: BMC Geriatr. 2021 Jul 1;21:406. doi: 10.1186/s12877-021-02326-3 (PMC8252309; doi:10.1186/s12877-021-02326-3)
Supplement: Supplementary file 14 — Additional file 14: Figure A3. Class membership probabilities by health class for the 1905 and 1910 cohorts considering only females. [file 12877_2021_2326_MOESM14_ESM.docx]

1. **Sensitivity analysis – considering only females in the LCA**

**Figure A3. Class membership probabilities by health class for the 1905 and 1910 cohorts considering only females.**

**
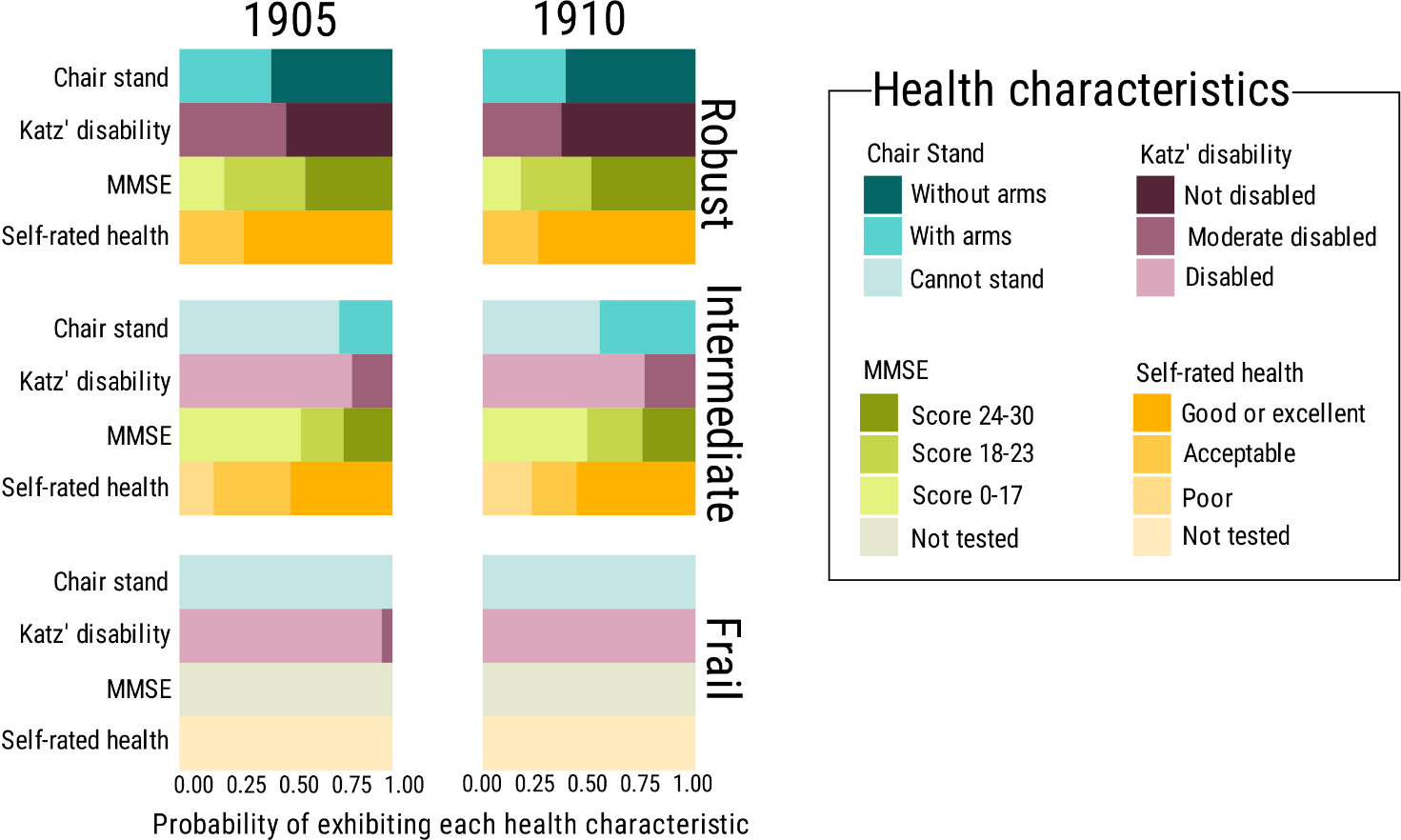
**
