# Supplementary material for: Stratification in health and survival after age 100: evidence from Danish centenarians
Source: BMC Geriatr. 2021 Jul 1;21:406. doi: 10.1186/s12877-021-02326-3 (PMC8252309; doi:10.1186/s12877-021-02326-3)
Supplement: Supplementary file 15 — Additional file 15: Figure A4. Survival probabilities above age 100 by health class and associated 95% confidence intervals for the 1905 and 1910 cohorts considering only females in the Latent Class Analysis. [file 12877_2021_2326_MOESM15_ESM.docx]

**Figure A4. Survival probabilities above age 100 by health class and associated 95% confidence intervals for the 1905 and 1910 cohorts considering only females in the Latent Class Analysis.**

**
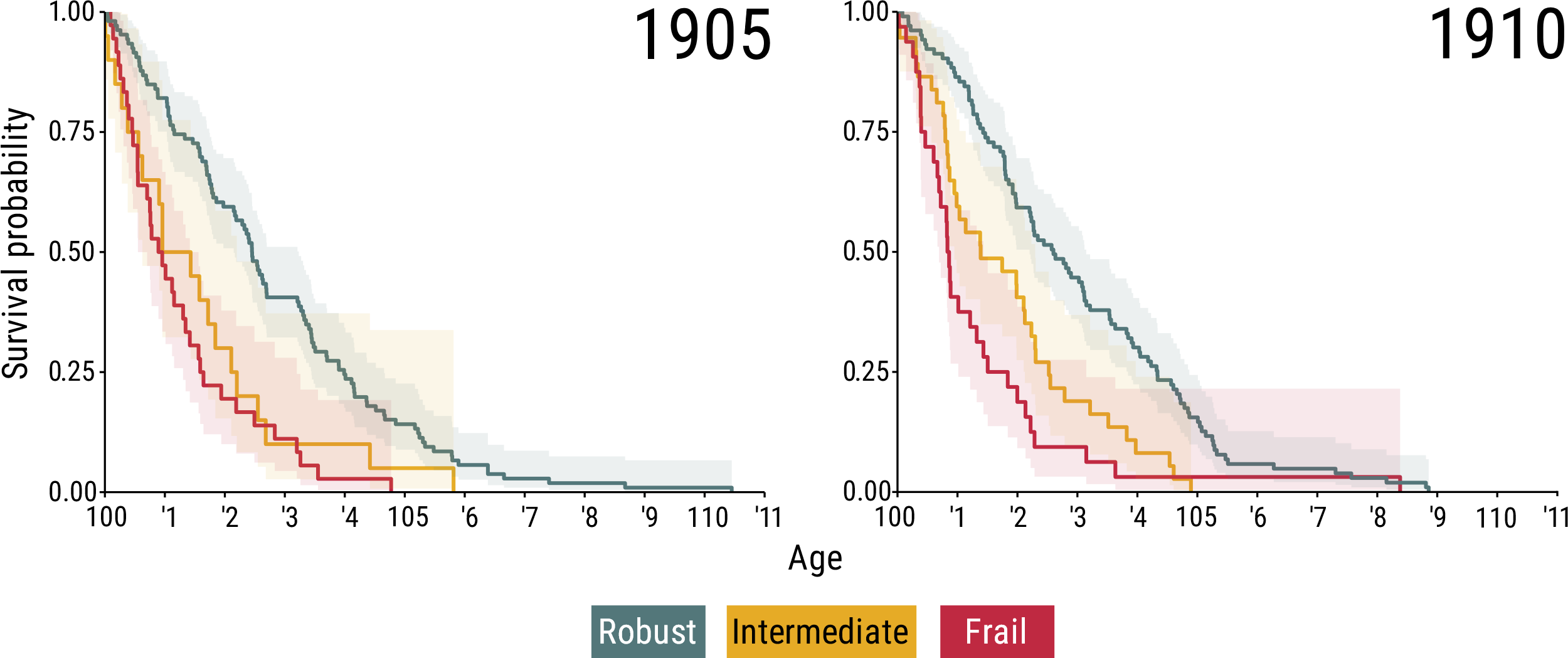
**
