# Supplementary material for: Stratification in health and survival after age 100: evidence from Danish centenarians
Source: BMC Geriatr. 2021 Jul 1;21:406. doi: 10.1186/s12877-021-02326-3 (PMC8252309; doi:10.1186/s12877-021-02326-3)
Supplement: Supplementary file 16 — Additional file 16: Table A12. Area under the curve by percentile for the 1905 and 1910 cohorts considering only females in the analysis. [file 12877_2021_2326_MOESM16_ESM.docx]

**Table A12. Area under the curve by percentile for the 1905 and 1910 cohorts considering only females in the analysis.**

|  |  | **1905 Cohort** | |  | **1910 Cohort** | |
| --- | --- | --- | --- | --- | --- | --- |
| **Percentile** |  | **Age** | **AUC** |  | **Age** | **AUC** |
| 95th |  | 105.61 | 0.64 |  | 105.72 | 0.65 |
| 96th |  | 105.95 | 0.68 |  | 106.08 | 0.65 |
| 97th |  | 106.26 | 0.68 |  | 106.39 | 0.64 |
| 98th |  | 106.95 | 0.68 |  | 107.09 | 0.64 |
| 99th |  | 107.94 | 0.68 |  | 108.15 | 0.62 |
|  |  |  |  |  |  |  |

Note: The AUC ranges from 0 to 1; a higher AUC implies a better prediction. Medford et al. define the frontier of survival as the 95th percentile of the centenarian age-at-death distribution. We included upper percentiles as a robustness check.
