# Supplementary material for: Stratification in health and survival after age 100: evidence from Danish centenarians
Source: BMC Geriatr. 2021 Jul 1;21:406. doi: 10.1186/s12877-021-02326-3 (PMC8252309; doi:10.1186/s12877-021-02326-3)
Supplement: Supplementary file 17 — Additional file 17: Figure A5. Class membership probabilities by health dimension for the 1905 and 1910 cohorts including Smoking in the Latent Class Analysis, both sexes. [file 12877_2021_2326_MOESM17_ESM.docx]

1. **Sensitivity analysis – including smoking behaviour**

**Figure A5. Class membership probabilities by health dimension for the 1905 and 1910 cohorts including Smoking in the Latent Class Analysis, both sexes.**

**
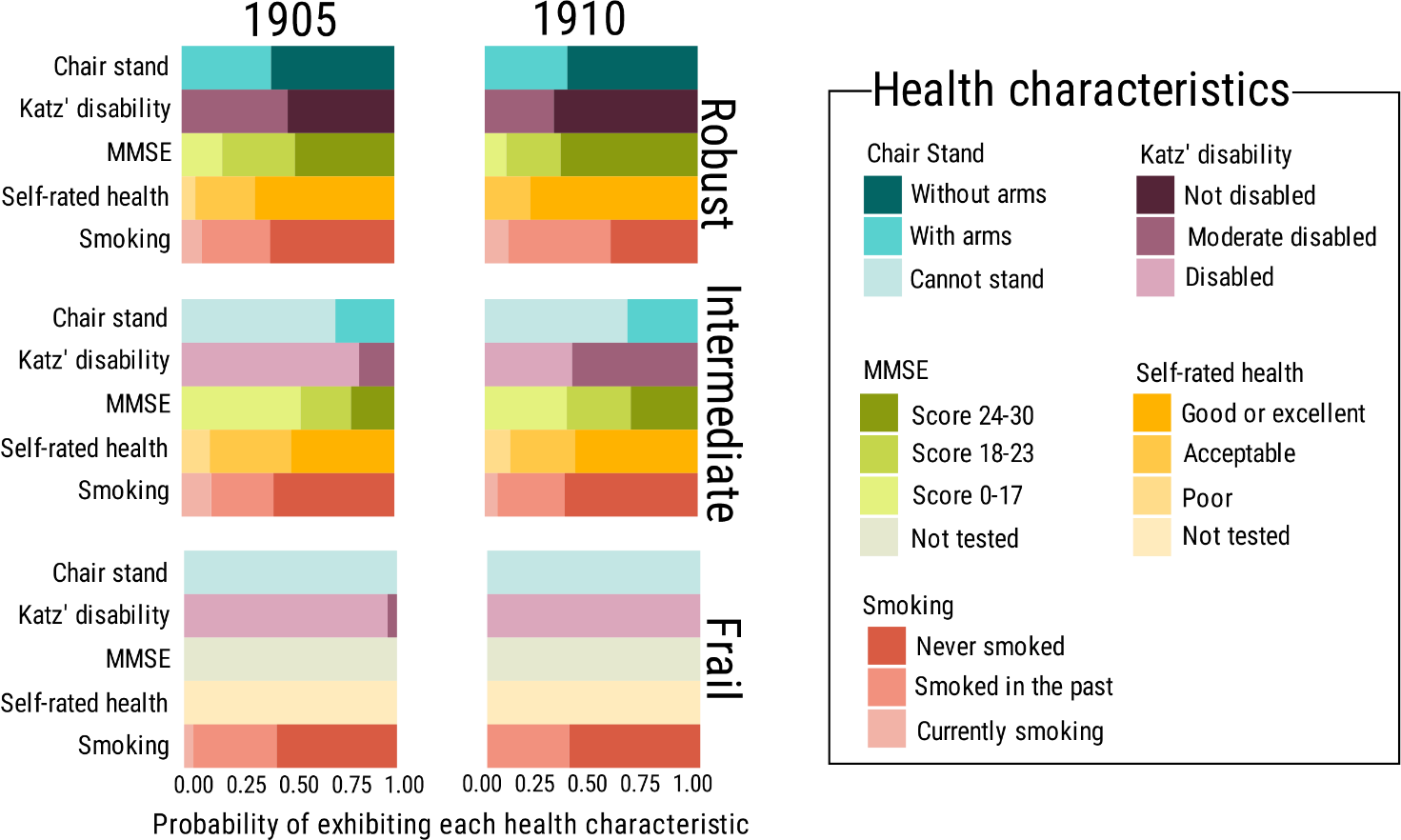
**
