# Supplementary material for: Stratification in health and survival after age 100: evidence from Danish centenarians
Source: BMC Geriatr. 2021 Jul 1;21:406. doi: 10.1186/s12877-021-02326-3 (PMC8252309; doi:10.1186/s12877-021-02326-3)
Supplement: Supplementary file 20 — Additional file 20: Figure A7. Class membership probabilities by health dimension for the 1905 and 1910 cohorts including only individuals with complete observations, without the creation of “no tested” category. [file 12877_2021_2326_MOESM20_ESM.docx]

1. **Sensitivity analysis – removing the missing data**

**Figure A7. Class membership probabilities by health dimension for the 1905 and 1910 cohorts including only individuals with complete observations, without the creation of “no tested” category.*
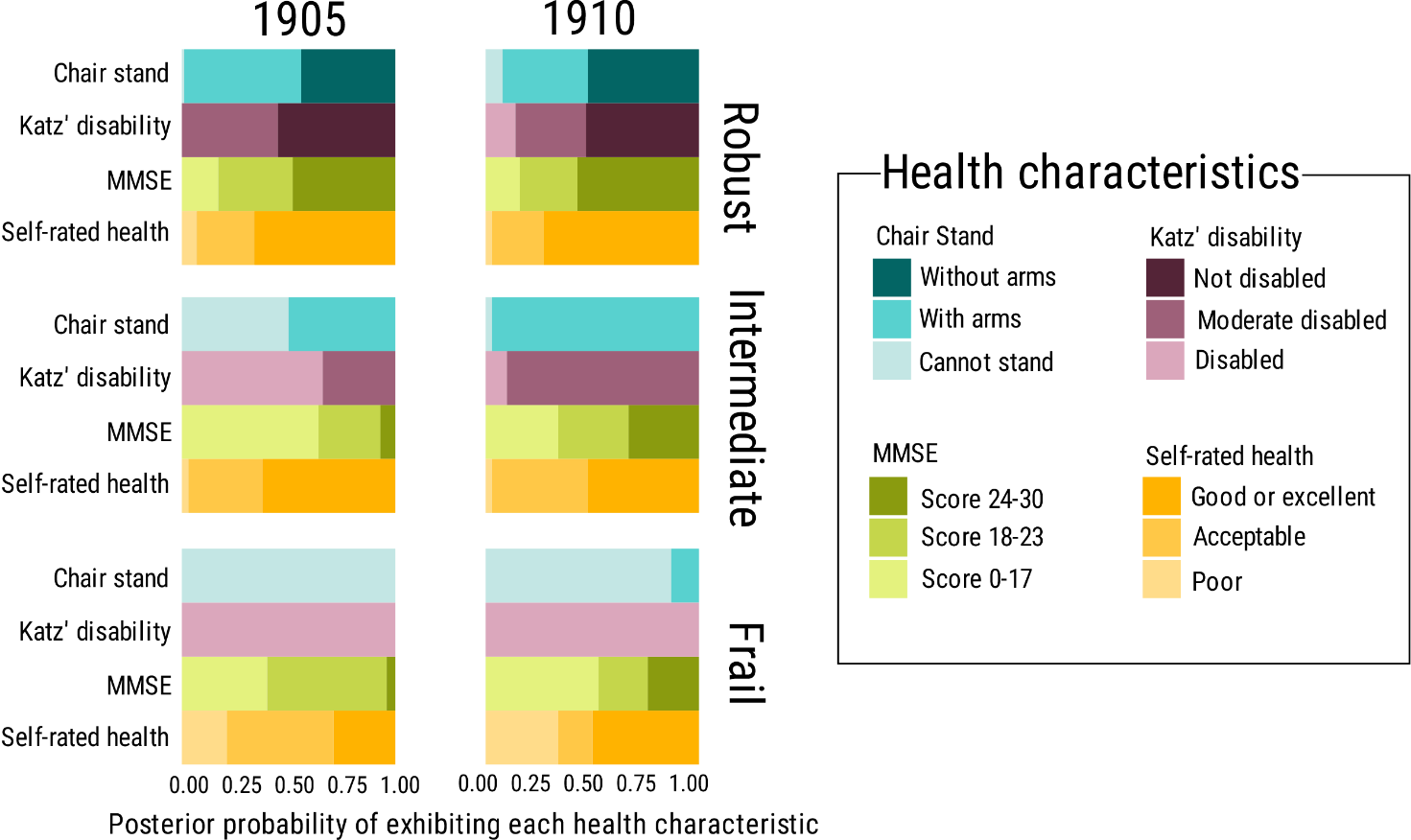
***
