# Supplementary material for: Stratification in health and survival after age 100: evidence from Danish centenarians
Source: BMC Geriatr. 2021 Jul 1;21:406. doi: 10.1186/s12877-021-02326-3 (PMC8252309; doi:10.1186/s12877-021-02326-3)
Supplement: Supplementary file 21 — Additional file 21: Figure A8. Class membership probabilities by health dimension for the 1905 and 1910 cohorts including those individuals that died before age 100 in the Latent Class Analysis, both sexes. [file 12877_2021_2326_MOESM21_ESM.docx]

1. **Sensitivity analysis – including those that died before age 100**

**Figure A8. Class membership probabilities by health dimension for the 1905 and 1910 cohorts including those individuals that died before age 100 in the Latent Class Analysis, both sexes.**

***
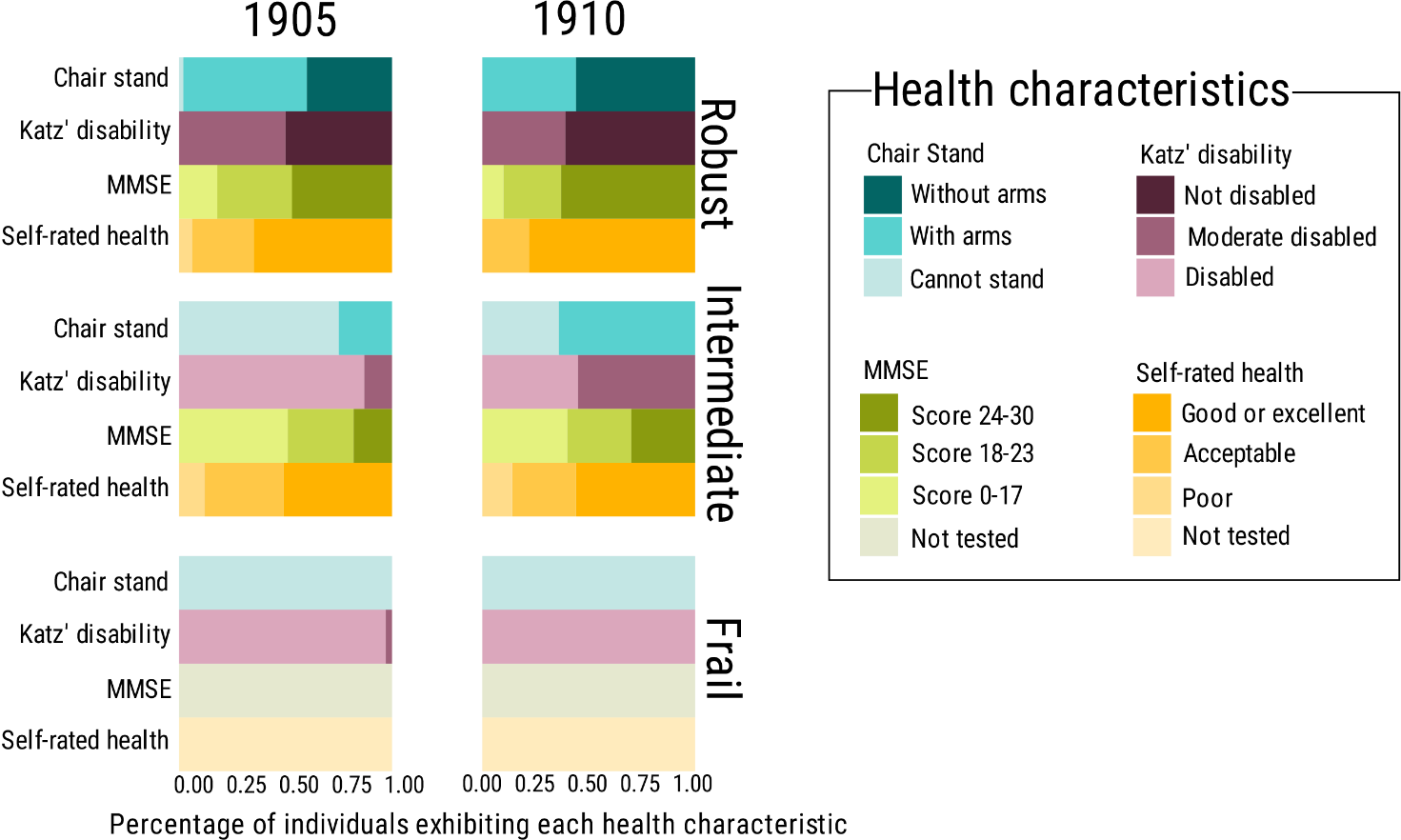
***
