# Supplementary material for: Stratification in health and survival after age 100: evidence from Danish centenarians
Source: BMC Geriatr. 2021 Jul 1;21:406. doi: 10.1186/s12877-021-02326-3 (PMC8252309; doi:10.1186/s12877-021-02326-3)
Supplement: Supplementary file 22 — Additional file 22: Table A14. Area under the curve by percentile for the 1905 and 1910 cohorts using Chair Stand (able to stand with and without hands) and MMSE (>24). [file 12877_2021_2326_MOESM22_ESM.docx]

1. **Calculation of AUC using only Chair Stand and MMSE as predictors of extreme survival as in Thinggaard et al (2016)**

Thinggaard et al (2016) found that Chair Stand combined with MMSE are good predictors of survival among Danish nonagenarians in the 1905 cohort. Specifically, those exhibiting MMSE greater than 28 and being able to stand up from their chair without hands are at greater chance to survive to age 100. Here we tested the predictive power of these characteristics for centenarians in the 1905 and 1910 cohorts. However, we softened the “robust threshold” to those that are able to stand with and without hands and have MMSE>24 given that only few individuals appear to have values of MMSE>28 and are able to stand from chair without hands. By using the softened version of the robust threshold, we are able to obtain similar predictive power (depicted by the AUC) to the one depicted in our LCA categorization. Both approaches are useful to predict the length of life in centenarians.

**Table A14. Area under the curve by percentile for the 1905 and 1910 cohorts using Chair Stand (able to stand with and without hands) and MMSE (>24)**

|  |  | **1905 Cohort** | |  | **1910 Cohort** | |
| --- | --- | --- | --- | --- | --- | --- |
| **Percentile** |  | **Age** | **AUC** |  | **Age** | **AUC** |
| 95th |  | 105.61 | 0.60 |  | 105.72 | 0.66 |
| 96th |  | 105.95 | 0.73 |  | 106.08 | 0.66 |
| 97th |  | 106.26 | 0.73 |  | 106.39 | 0.65 |
| 98th |  | 106.95 | 0.71 |  | 107.09 | 0.65 |
| 99th |  | 107.94 | 0.72 |  | 108.15 | 0.61 |
|  |  |  |  |  |  |  |

Note: The AUC ranges from 0 to 1; a higher AUC implies a better prediction. Medford et al. define the frontier of survival as the 95th percentile of the centenarian age-at-death distribution. We included upper percentiles as a robustness check. The AUC in this table was calculated using only Chair Stand and MMSE as in Thinggaard et al, (2016)
